# Extremal events dictate population growth rate inference

**DOI:** 10.1371/journal.pcbi.1014088

**Published:** 2026-05-13

**Authors:** Trevor GrandPre, Ethan Levien, Ariel Amir

**Affiliations:** 1 Department of Physics, Washington University in St. Louis, St. Louis, Missouri, United States of America; 2 National Institute for Theory and Mathematics in Biology, Northwestern University and The University of Chicago, Chicago, Illinois, United States of America; 3 Department of Physics, Princeton University, Princeton, New Jersey, United States of America; 4 Department of Mathematics, Dartmouth College, Hanover, New Hampshire, United States of America; 5 Department of Physics of Complex Systems, Weizmann Institute of Science, Rehovot, Israel; University of Helsinki, FINLAND

## Abstract

Recent methods have been developed to map single-cell lineage statistics to population growth. Because population growth selects for exponentially rare phenotypes, these methods inherently depend on sampling large deviations from finite data, which introduces systematic errors. A comprehensive understanding of these errors in the context of finite data remains elusive. To address this gap, we study the error in growth rate estimates across different models. We show that under the usual bias-variance decomposition, the bias can be decomposed into a finite-time bias and nonlinear averaging bias. We demonstrate that finite-time bias, which dominates at short times, can be mitigated by fitting its monotonic behavior. In contrast, at longer times, nonlinear averaging bias becomes the predominant source of error, leading to a phase transition. This transition can be understood through the Random Energy Model, a mean-field model of disordered systems, where a few lineages dominate the estimator. Applying these methods to experimental data demonstrates that correcting for biases in lineage-based approaches yields consistent results for the long-term growth rate across multiple methods and enables the reverse-engineering of dynamic models. This new framework provides a quantitative understanding of growth rate estimators, clarifies the conditions under which they can be effectively applied to finite data, and introduces model-free approaches for studying the connections between physiology and cell growth.

## Author summary

Understanding how quickly a microbial population grows is a central question in biology, intimately linked to evolutionary fitness. While recent advances have made it possible to estimate growth rates from single-cell data, these estimates often vary widely in practice. In this work, we demonstrate that such inconsistencies arise from fundamental limitations imposed by the fact that exponential growth selects for exponentially rare phenotypes, which dictate the growth rate. Here, we show that two widely used “model-free” approaches both suffer from tradeoffs between two sources of bias: at short timescales, limited observation windows bias estimation, while at longer timescales finite sampling fails to adequately capture the exponentially rare trajectories required for convergence of the exponential average. We present a unified framework that disentangles and corrects both sources of error, enabling robust growth rate estimates even from modest datasets. Our results clarify when lineage-based methods can be trusted and what kinds of data are required to accurately infer population growth from single-cell measurements.

## 1. Introduction

A central objective in biology is to understand the relationship between genotype, phenotype, and fitness [1–10]. In the context of microbes, a key component of fitness is the long-term growth rate [7], defined as


$$\begin{array}{c} \Lambda =\lim_{T\to \infty }\frac{1}{T}\ln\left[\frac{N(T)}{N(0)}\right], \end{array}$$ (1)

where *N*(0) and *N*(*T*) are the population sizes at times *t* = 0 and *t* = *T*.

Experimentally, Λ can be measured by bulk fitness assays. When performed on libraries of different strains, such experiments can yield insight into the genotype-to-fitness map [11]. However, they do not reveal how individual single-cell traits contribute to fitness. The theoretical foundations of this question can be traced back to seminal work in demography, dating to Euler [12]. Most notably, the Euler-Lotka equation relates the population growth rate to the distribution of individual lifetimes within a population. For a population undergoing binary fission, this relationship is expressed as [12–15]


$$\begin{array}{c} E[e^{-\Lambda \tau }]=\frac{1}{2}\;. \end{array}$$ (2)

In its modern form [16–18], which accommodates correlated generation times, the average E[·] is taken over all generation times τ throughout the entire population tree. A key implication of Eq. 2 is that


$$\begin{array}{c} \Lambda \geq \frac{\ln(2)}{E[\tau ]} \end{array}$$ (3)

which follows from Jensen’s inequality. In a population where every cell divides after a fixed time $\tau_{0}$, the growth rate is exactly $\ln(2)/\tau_{0}$. Equation (2) can be used to show that, for a fixed mean of the generation-time distribution, increasing its variability increases the population growth rate [19]. However, variability in single-cell growth rates, while fixing the mean growth rate, may lower the population growth rate compared to a population without such variability [18,20]. In this case, the inequality in Eq. (3) still holds, but the impact of growth rate fluctuations is captured through changes in E[τ], leading to a slower population growth rate.

**Fig 1:**
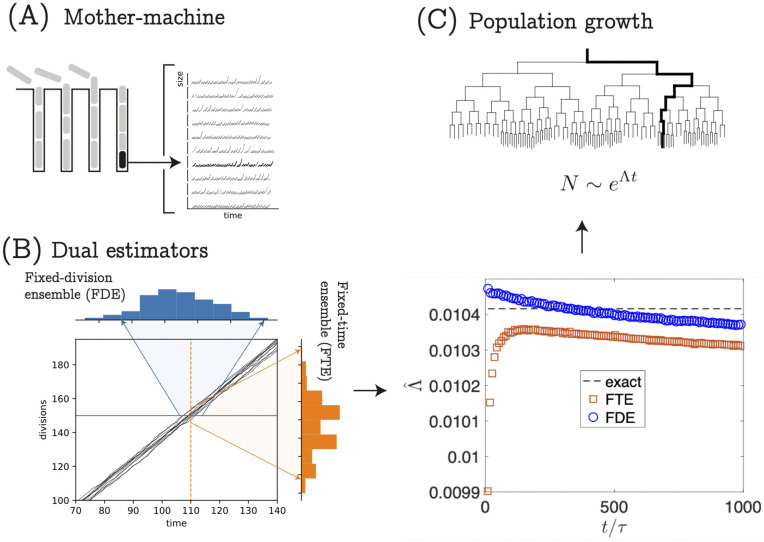
(A) Schematic of the mother-machine microfluidic device used to collect single-cell lineage data, alongside an example of lineage data. (B) Dual ensembles can be used to extract large-deviation statistics relevant for growth-rate inference. This is illustrated using data generated from simulations of the cell-size control model defined in S1 Text, Cell-size control model [21], which produce an ensemble of single-cell trajectories relating division number to elapsed time. The blue histogram is obtained by taking a horizontal cross-section of this data at 150 divisions, while the orange histogram is obtained by taking a vertical cross-section at fixed elapsed time. Using these histograms to estimate the scaled cumulant generating function yields the fixed-divisions ensemble (FDE), Eq. (5), in which the number of divisions is fixed and the elapsed time fluctuates, and the fixed-time ensemble (FTE), Eq. (4), in which the total time is fixed and the number of divisions fluctuates, as defined in the SI [21]. (C) These lead to estimators of the exponential growth rate of a growing population whose lineages, when sampled by traveling forward in time from the root of the tree, have the same division statistics as the lineages in (A). The plot below the population tree shows the estimators applied to the same model of cell-size control as in (B).

Mother machine experiments offer a powerful investigative tool to explore the effects of mutations on single-cell lineage dynamics (see Fig 1A). In these experiments, cells are confined in microfluidic channels, with all but one lineage being expelled from the experiment in each channel [22,23]. Barcoding techniques have been developed to enable mother machine assays across multiple strains simultaneously [24]. A key question is how to connect the data from these experiments to data from bulk fitness assays. Although the Euler-Lotka equation provides a theoretical framework for connecting single-cell generation time statistics to bulk fitness, the average in Eq. 2 is taken over the entire population tree, whereas mother machines capture data from only single lineages. These two approaches coincide only when generation times are uncorrelated.

Recently, studies have explored the challenges in predicting the population growth rate from single-cell lineage statistics [5,10,20,25–27]. One estimator of the population growth rate from lineages is described in Ref. [25]:


$$\begin{array}{c} {\hat{\Lambda }}_{1}=\frac{1}{T}\ln\left[\frac{1}{M}\sum_{i=1}^{M}2^{N_{t}^{\left(i\right)}}\right]\; \end{array}$$ (4)

where $N_{t}^{\left(i\right)}$ is the number of divisions along the i’th lineage within a fixed time *T* and *M* is the number of lineages (see Methods 4.1 for more details). In practice, this method is sensitive to extremal statistics of the sampled generation times or division counts, leading to non-monotonic convergence in the duration of time intervals. At longer times (and fixed *M*), the sample averages become dominated by extremal statistics. As a result, the naive estimate on the right-hand side of Eq. (3) is obtained rather than the true growth rate [25].

Another estimator of the population growth rate from lineages is described in Ref. [26]:


$$\begin{array}{c} \frac{1}{n}\ln\left[\frac{1}{M}\sum_{i=1}^{M}e^{-T_{n}^{\left(i\right)}{\hat{\Lambda }}_{2}}\right]=-\ln(2)\;. \end{array}$$ (5)

Here, $T_{n}^{\left(i\right)}$ represents the time of each lineage at a fixed number of divisions *n*, and we can solve for ${\hat{\Lambda }}_{2}$ in the exponent. Unlike the standard Euler-Lotka equation in Eq. (2), this formulation is a generalized Euler-Lotka equation tailored to observables along lineages, making it particularly suitable for mother-machine data.

Both of these estimators can be related to a statistical physics theory called large deviation theory (see Methods 4.1), a generalization to the equilibrium free energy. Within this framework, they are considered to be two ensembles. Equation 4 is to be considered a fixed-divisions ensemble (FDE) and Eq. 5 is a fixed-time ensemble (FTE). In the limit that the time *T* goes to infinity in the FTE and *n* goes to infinity in the FDE (e.g., an infinite amount of data), both estimators match ${\hat{\Lambda }}_{1}={\hat{\Lambda }}_{2}$. However, experimental data is finite, which leads to inconsistencies between the two estimates in practice. Our understanding of how these two methods perform in practice with finite data is incomplete and qualitative.

In this paper, we quantitatively study the systematic biases in both estimators, and show how to remove these biases to accurately compare the estimators. In Sec. 2.1, we quantify the biases using the conventional bias-variance trade-off framework [28,29]; the bias can be decomposed into two distinct components: a finite-time bias that persists even with an infinite number of lineages, and a nonlinear averaging bias arising when the lineage ensemble is not self-averaging (see Methods 4.1). In Section 2.2, we show that finite-size scaling—a well-established concept in statistical physics [30]—can be employed to completely eliminate the finite-time bias.

In Section 2.3, we establish that finite-lineage effects can be understood through their connection to the Random Energy Model (REM), a mean-field model of disordered systems. This connection clarifies how non-monotonic convergence arises from a phase transition into a “frozen state”. In the REM, this transition corresponds to the system entering a low-temperature phase dominated by a few energy states. Similarly, in the context of growth-rate estimators, this transition occurs when a few extremal lineages begin to dominate the ensemble, leading to analogous behavior. Our analysis draws parallels with previous studies on Jarzynski estimators of free energy differences [31–33], and also connects to research on estimating large deviations, particularly in the context of predicting the bandwidth of tele-traffic streams [34–36]. We explore these connections further in the SI [21]. Our findings provide a framework for determining when growth rate estimation is feasible from single-cell data.

## 2. Results

### 2.1. Error decomposition

The performance of the estimators can be quantified by root mean square error,


$$\begin{array}{c} \varepsilon (\hat{\Lambda })=\sqrt{E[(\hat{\Lambda }-\Lambda {)}^{2}]}. \end{array}$$ (6)

Assuming perfect measurements of division times and counts, applying the standard bias-variance decomposition yields [28,29]:


$$\begin{array}{c} \begin{array}{cc} \varepsilon^{2}(\hat{\Lambda }) & =\underset{var(\hat{\Lambda })}{\underbrace{E[(\hat{\Lambda }-E[\hat{\Lambda }]{)}^{2}]}}+\underset{Bias(\hat{\Lambda }{)}^{2}}{\underbrace{(E[\hat{\Lambda }]-\Lambda {)}^{2}}} \end{array} \end{array}$$ (7)

where E[·] is an ensemble average of $\hat{\Lambda }$ from many realizations of the growth process. This formula follows from the definition of var and $\varepsilon (\hat{\Lambda })$.

To fully grasp the non-monotonic convergence, it is crucial to closely examine the bias term, which can be further decomposed as:


$$\begin{array}{c} Bias(t,M)=\underset{{Bias}_{nl}(t,M)}{\underbrace{\left(E[\hat{\Lambda }]-\Lambda (t)\right)}}+\underset{{Bias}_{ft}(t)}{\underbrace{\left(\Lambda (t)-\Lambda \right)}} \end{array}$$ (8)

where $\Lambda (t)=t^{-1}\ln E[2^{N_{t}}]$ and $\Lambda =\lim_{t\to \infty }\Lambda (t)$. Note that by taking the expectation, we assume the large *M* limit has been reached, with fixed lineage durations or division counts for the FTE and FDE, respectively. However, in order to compute $\varepsilon (\hat{\Lambda })$ from Eq. (6) and its different contributions from Eq. (7) using simulations, we must replace all averages by their sample averages over many realizations of the growth process, as detailed in Eqs. (26) and (30).

The nonlinear averaging bias, Bias_nl_(*t*, *M*), is intimately connected to the concept of *quenched free energy* in disordered systems, a relationship that emerges when $\langle \cdot \rangle_{M}$ is interpreted as a partition function. This connection will be clarified in Section 2.3, where we introduce a scaling of lineage durations with *M*, analogous to the approach used in [31,32] to estimate free energy differences. It is important to note that the dependence on *M* arises solely from $var(\hat{\Lambda })$ and Bias_nl_, and both go to zero in the limit that *M* goes to infinity. Hence, the only error at large *M* is the finite-time error:


$$\begin{array}{c} \lim_{M\to \infty }\varepsilon^{2}(\hat{\Lambda })={Bias}_{ft}(t) \end{array}.$$ (9)

In the next section, we will present numerical and theoretical results and explore the differences in the finite-time bias between the FTE and FDE.

#### 2.1.1. Numerical results.

We now present numerical results that highlight the key features of the different terms in the error expression. We conduct simulations using a simple model where generation times follow a first-order autoregressive process (AR(1)) [18,20,37]:


$$\begin{array}{c} \tau_{n+1}=\tau_{0}(1-c)+\tau_{n}c+\xi_{n}, \end{array}$$ (10)

where $\tau_{0}$ is the average generation time, $\tau_{n}$ is the parent generation time, *c* is the Pearson correlation coefficient between parent and offspring, and $\xi_{n}$ represents noise with zero mean and a variance given by $\sigma_{\xi }^{2}=(1-c^{2})\sigma_{\tau }^{2}$. This model incorporates correlations between generation times, which are essential for maintaining homeostasis, and captures key aspects of the convergence for the FTE and FDE estimators.

The long-time population growth rate for the model in Eq. (10) was first calculated in Ref. [20] to be


$$\begin{array}{c} \Lambda =\frac{2\ln(2)/\tau_{0}}{1+\sqrt{1-2\ln(2)(\sigma_{T}/\tau_{0}{)}^{2}}} \end{array}$$ (11)

where


$$\begin{array}{c} \sigma_{T}^{2}=\sigma_{\tau }^{2}\frac{\left(1+c\right)}{\left(1-c\right)}\;. \end{array}$$ (12)

An alternative derivation of the population growth rate of this model using a *Second Cumulant Expansion* of the FDE is discussed in Methods 4.2.

**Fig 2:**
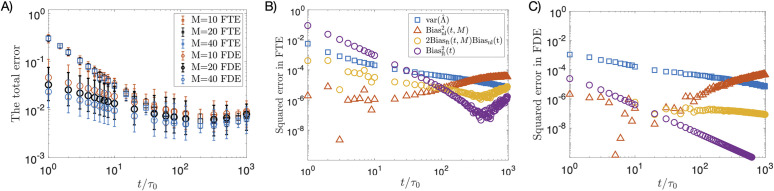
Systematic error in growth rate estimates. Simulations were done using the model in Eq. (10) with parameters given in Sec. 2.1.1. The analytical solution of the long-time population growth rate of this model is shown in Eq. 11. **(A)** The average total error for both estimators (Eq. (6)) for *M* = 10, *M* = 20, and *M* = 40 lineages over 10^3^ generations, calculated from 10^3^ realizations, with error bars representing the standard deviation. **(B)** The absolute squared error (Eqs. (7) and (8)) and all contributions for the FTE. **(C)** The absolute squared error (Eqs. (7) and (8)) and all contributions for the FDE.

In Fig 2A, we show the total error for the FTE and FDE from Eq. (6) from simulations over $t/\tau_{0}=10^{3}$ generations. The parameters used were $\tau_{0}=1$ and $\sigma_{\tau }=0.2$, with 10, 20, and 40 lineages. Note that the error decreases over time for both methods, but the FDE has an order of magnitude smaller error for the first 100 generations. The methods have comparable error after the first 100 generations.

Next, we look at the different contributions to error. In Fig 2B and Fig 2C, we show the four contributions to the total error based on Eq. (7) for the FTE and FDE, respectively. At short times, the FTE estimator is dominated by the finite-time bias, while the FDE estimator is dominated by the variance of the estimator, though it retains a small, nonzero finite-time bias. For both methods, the variance of the estimators tends to zero as the number of generations tends to infinity, making the total error primarily driven by the nonlinear bias, Bias_nl_(*t*, *M*).

In the next section, we will discuss the monotonic convergence of Bias_ft_(*t*) for both methods and why the FDE has a much smaller error than FTE. Then, in Sec. 2.3 we will discuss the nonlinear averaging bias, Bias_nl_(*t*, *M*).

### 2.2. Finite duration bias (Bias_ft_(*t*))

In this section, we examine the behavior of the finite time and finite division number error in both the FTE and FDE. Specifically, we demonstrate that both ensembles exhibit inverse scaling with lineage duration given that the lineage number is large enough, while providing a justification for the significantly smaller prefactor observed in the FDE.

#### 2.2.1. Inverse time scaling.

In previous work [25], we demonstrated that for small noise in generation times, ${\hat{\Lambda }}_{1}$ converges inversely with lineage length, as described by


$$\begin{array}{c} {Bias}_{ft}(t,M)=\frac{A}{t}+o\left(\frac{1}{t}\right)\;. \end{array}$$ (13)

Generally, deriving an exact expression for the finite-time bias is challenging, even when the large deviations are well understood. However, as we discuss below, in certain cases, this term can be approximately removed from the estimator using a finite-time scaling approach.

In this work, we demonstrate that a similar result can be obtained for the FDE estimator. We can compute the convergence of the FDE estimator exactly for all times for the model in Eq. (10) (see Methods 4.1 and S1 Text, Derivation of finite time growth rate for the FDE [21] for derivations). The leading order correction to the FDE will be


$$\begin{array}{c} {\hat{\Lambda }}_{2}=\Lambda +\frac{B}{n}+o\left(\frac{1}{n}\right)\;, \end{array}$$ (14)

where *B* is the finite-time coefficient. In practice, the coefficients for both FDE and FTE are determined directly from the data by fitting the coefficients *A* and *B* at short times.

**Fig 3:**
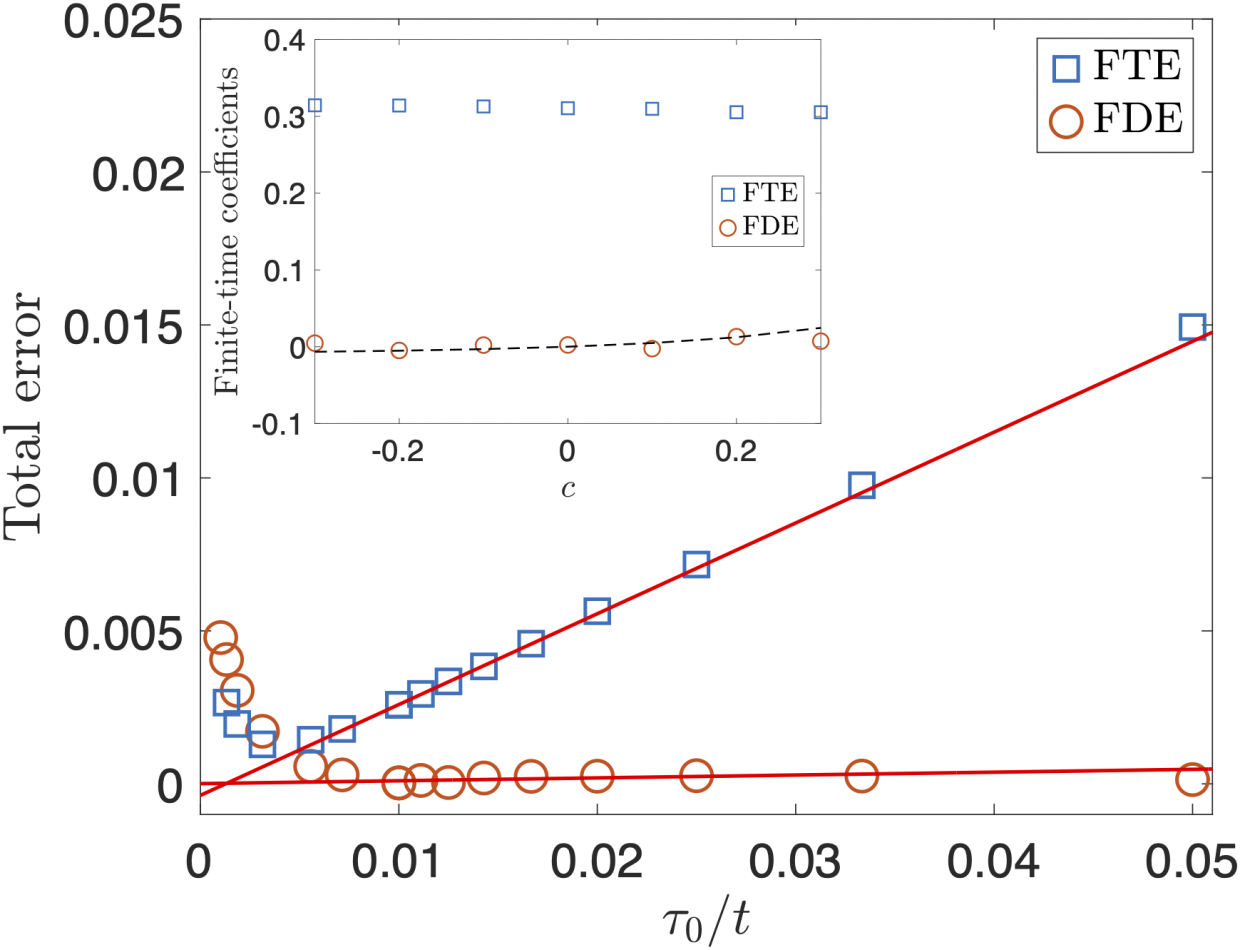
The finite-time coefficients for the model in Eq. 10. The linear trend from right to left (red lines) is due to the finite-time error (see Eqs. (13) and (14)). The blue squares and red circles represent data from the FTE and FDE, respectively, obtained from 1,000 simulations using the same parameters as Fig 2. Error bars are omitted because they are significantly smaller than the symbols. For the FDE, the x-axis represents the number of divisions, $n=\tau_{0}/t$. The inset shows the finite-time coefficients for a range of correlation coefficients. The black dotted line is the prediction of the FDE (see SI Text, Derivation of finite-time growth rate for the FDE [21]). At larger times, the total error is no longer linearly decreasing in time so the fit is stopped when the error becomes nonconvex.

We demonstrate this in Fig 3 for the model in Eq. (10) and show the fitting procedure and the finite-time coefficients as a function of the correlation strength, *c*. The finite-time coefficients are obtained by plotting the total error vs. $\tau_{0}/t$ and extracting the slope at small times. In this format, fitting the linearly decreasing points from right to left gives the finite-time coefficients. As the number of generations gets too large, the nonlinear averaging bias dominates and the error becomes non-convex and begins to increase again. At the point that the error begins to increase, we stop the fitting. If there were an infinite amount of lineages, the estimators would linearly decrease until zero.

In the inset of Fig 3, we show the value of the finite-time coefficients over a range of *c* values. We find that the coefficient for the FDE is consistently about 10 times smaller than that of the FTE in the SI Text, Transport equation derivation of FDE [21], we present a derivation using the von Foerster approach to explain this disparity. The key point is that the FTE exhibits a finite-time correction regardless of the initial conditions or the presence of correlations between generation times. In contrast, the FDE coefficient can vanish in the limit where such correlations are absent for certain initial conditions.

As shown in Fig 3, there is a finite-time bias for both FTE and FDE, which decreases monotonically with time initially. At longer times, however, the total error begins to increase nonlinearly. This behavior arises because the nonlinear averaging bias starts to dominate the total error. The phenomenon of non-monotonic convergence for the FTE was first identified in Ref. [25], where it was observed that, for large times and a fixed number of lineages, the estimator approaches the naive estimate on the right-hand side of Eq. (3). However, previous work did not quantitatively characterize the transition from monotonic to non-monotonic convergence in the total error for the FTE, and this transition has not been observed before for the FDE.

Next, we demonstrate that the crossover from monotonic to non-monotonic convergence in the total error represents a second-order phase transition. Furthermore, we quantitatively show how this systematic error can be avoided.

### 2.3. Nonlinear averaging bias (Bias_nl_) and connection to the Random Energy Model

Here we discuss the bias resulting from the finite number *M* of lineages. Inspired by the approach in Refs. [31,32], we obtain an approximate expression for the bias in the FDE using a known formula for the free energy density of the REM.

As discussed in Methods 4.1 and demonstrated by our numerical results in Sec. 2.1.1, the empirical averages used to estimate the scaled cumulant generating functing (SCGF) are influenced by a linearization effect, a phenomenon described in Ref. [33]. This effect is analogous to the error seen in Jarzynski’s Equality estimators of free energy differences [31], which can be understood through a connection to the Random Energy Model (REM). The REM is a mean-field model for disordered systems, which assumes that the energies of each state are sampled independently from a Gaussian distribution [38]. In this model, the partition function *Z*_*N*_ is simply an iid sum:


$$\begin{array}{c} Z_{N}=\sum_{i=1}^{2^{N}}e^{-\beta \sqrt{N/2}X_{i}}, \end{array}$$ (15)

where *X*_*i*_ are iid standard normal variables which would represent energy states in REM, but will represent individual lineages in our context. Additionally, within the original REM, *N* and β would be the system size and the inverse temperature. As we show below, in our case the *N* is related to the logarithm of the number of lineages and β is related to the ratio of the logarithm of the number of lineages to the number of divisions. Consequently, many properties of the REM can be derived using classical extreme value theory, without resorting to the more complex mathematical tools often required in the study of disordered systems (In many statistical mechanics papers, *X*_*i*_ are taken to have a variance of 1/2. Therefore, we have introduced a factor of 1/2 in the scaling of *N* to align our definition of the inverse temperature with the standard literature.). The free energy density of the model is given by


$$\begin{array}{c} f_{N}(\beta )=-\frac{1}{\beta N}\ln Z_{N}, \end{array}$$ (16)

and has an exact solution in the thermodynamic limit (see Methods 4.3).

We begin by examining the connection of REM to the FDE, which offers two key advantages. First, in this context, we can approximate $T_{n}^{\left(i\right)}$ using a Gaussian distribution, whereas for the FTE, we must contend with the counting variables $N_{t}^{\left(i\right)}$. Second, the finite-time bias in the FDE is minimal and can be effectively eliminated in our simulations by setting the mother-daughter correlations to zero, thereby allowing us to isolate Bias_nl_.

The goal in this section is to understand when the system will not converge to the correct population growth rate due to the nonlinear averaging bias. We use the AR(1) process defined in Eq. (10) for the simulations in this section. We express the exponent of Eq. (30) as


$$\begin{array}{c} zT_{n}^{\left(i\right)}=z\tau_{0}n+z\sigma \sqrt{n}X_{i}, \end{array}$$ (17)

where *X*_*i*_ follows a standard normal distribution (It is important to note that this expression is valid only when the coefficient of variation, *CV*_*T*_, is sufficiently small, ensuring that the likelihood of negative times remains negligible.). Next, we set *M* = 2^*N*^ and fix


$$\begin{array}{c} \alpha_{FDE}=\sqrt{\frac{2n}{N}}. \end{array}$$ (18)

The free energy for the REM has an exact solution in the thermodynamic limit, but we cannot solve for $\hat{\Lambda }$ analytically, since there is no closed-form expression for the finite-size free energy density. However, by approximating the finite-size free energy with its thermodynamic limit expression, the resulting equation can be solved to yield an estimate of the FDE estimator:


$$\begin{array}{c} {\hat{\Lambda }}_{REM}(\alpha )=\left\{ \begin{array}{cc} \Lambda & \alpha <\alpha_{c}\\ \frac{2\ln(2)(1-\alpha^{2}/2)}{\tau_{0}(2\alpha \sigma /\tau_{0}\sqrt{\ln(2)}-\alpha^{2})} & \alpha \geq \alpha_{c} \end{array}\right. \end{array}$$ (19)

with the critical value of α given by


$$\begin{array}{c} \alpha_{c}=\frac{2\sqrt{\ln(2)}}{\Lambda \sigma }\;. \end{array}$$ (20)

Here Λ is given by Eq. (11). Note that a useful approximation to $\alpha_{c}$ is


$$\begin{array}{c} \alpha_{c}\approx 2/(\sqrt{\ln(2)}{CV}_{T})\;. \end{array}$$ (21)

It can be checked that ${\hat{\Lambda }}_{REM}(\alpha )$ is indeed continuous at $\alpha_{c}$ and as α→∞ tends to $\ln(2)/\tau_{0}$ since the transition to the frozen state is second order.

**Fig 4:**
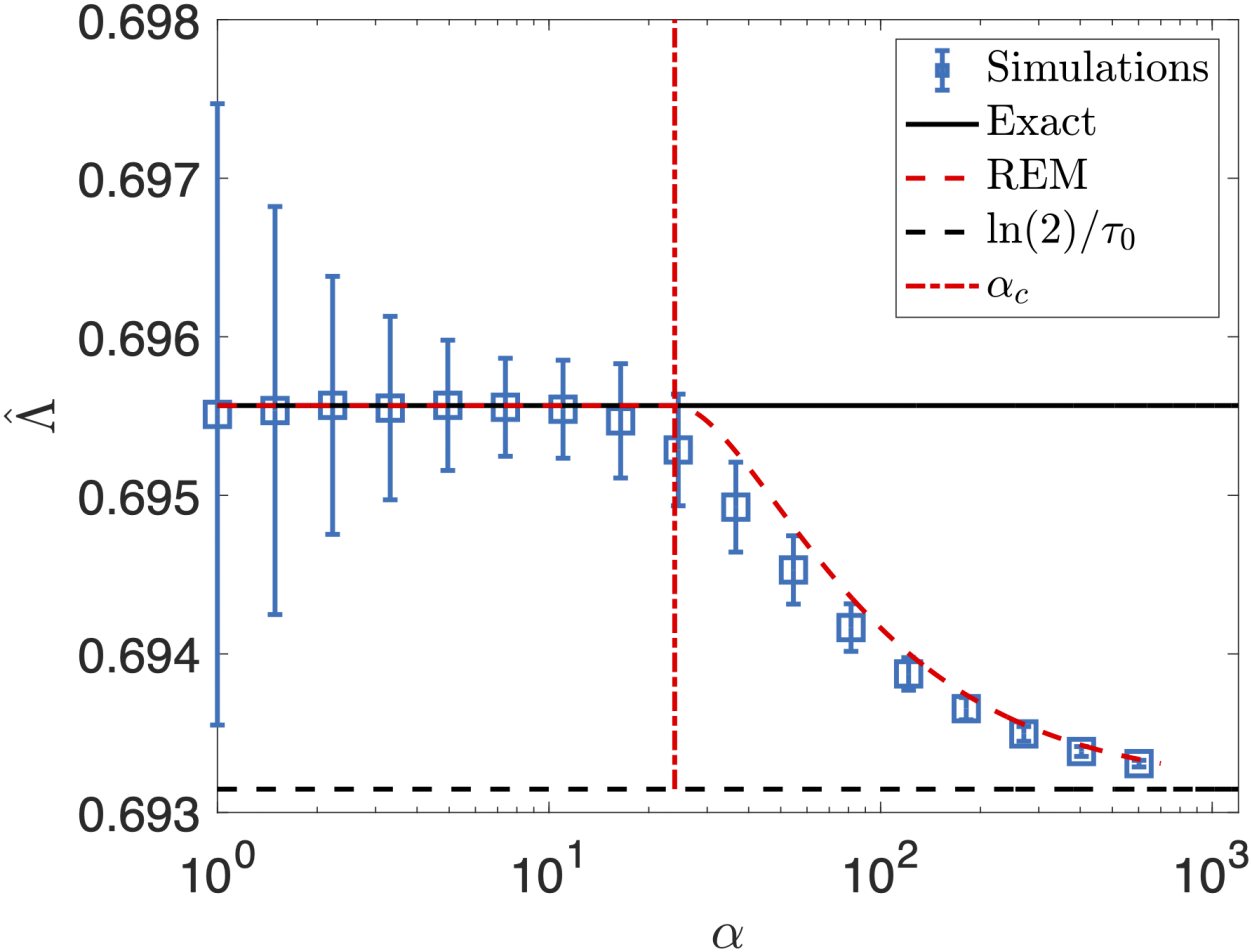
Estimates of Λ using the FDE compared to the theoretical prediction of the Random Energy Model (Eq. (45)). The blue squares represent the mean of 1,900 realizations, with error bars indicating the standard deviation. These error bars monotonically decrease as α increases, due to the monotonically decreasing variance term in the first term on the right-hand side of Eq. 7. Simulations are performed with independent Gaussian generation times with σ=0.1 and *c* = 0. Different values of α were realized by fixing *M* and modulating *n*. The black solid line is the exact population growth rate. The red dotted line is the prediction from Eq. 45, the dotted black line is $\ln(2)/\tau_{0}$, and the vertical red dashed-dotted line represents $\alpha_{c}$ (Eq. (46)).

In Fig 4, we show the phase transition as a function of $\alpha_{FDE}$ for the FDE from simulations with *c* = 0, τ=1, and σ=0.1. Small $\alpha_{FDE}$ corresponds to the high temperature regime and large α would be analogous to a low-temperature regime which is predicted by Eq. (20). As the finite-time bias vanishes with n→∞, Eq. (20) provides insight into when Bias_nl_ begins to increase, and thereby how small we need α to be in order to obtain reliable growth rate estimates. Indeed, our numerical experiments demonstrate that the REM-based theory effectively captures the transition in Bias_nl_. In addition, the error bars decrease as α increases because the variance between realizations—described by the first term on the right-hand side of Eq. 7—monotonically decreases over time. In Methods 4.3, we show that, similar to the FDE, the FTE also quantitatively agrees with the REM framework. However, the decay after the phase transition differs slightly between the two ensembles.

As seen in Fig 4, the analytical solution is slightly different on the right of the transition. Most likely, the deviations from the exact value seen in Fig 4 are due to finite-size effects. For a given α, both *n* and ln(M) should go to infinity while leaving the ratio n/lnM fixed. Deviations from REM were also seen in sampling Jarzynski’s Equality [31] (see SI Text, Connection to other work).

### 2.4. Application of estimators on real data

Now that we have an understanding of the convergence for both methods, we can apply this to experimental data. The ideal case is to have as long lineages as possible and avoid the linearization effect. Then, the estimators only have finite time bias, which can be fitted and corrected. We can find how long lineages need to be to avoid the linearization effect for a given lineage size by using the equation for the $\alpha_{c}$ in Eq. (20). For a given value for *M*, we can find the value of time durations before the nonlinear averaging bias takes over from the inequality:


$$\begin{array}{c} n_{c}<\frac{\ln(M)\alpha_{c}^{2}}{2\ln(2)}\;. \end{array}$$ (22)

We would parse the data so that the number of divisions is below *n*_*c*_, for a given lineage number.

We consider *E. coli* grown at 25 °C with data from Ref. [23]. The data contains 70 lineages with about 70 generations each. Hence, using Eq. 22 with *M* = 70 and $\alpha_{c}\approx 7$, we find that the linearization effect can be avoided if *n* < 150. To accurately extract the long-time population growth rate, we apply both methods to the lineage data and use the finite-time coefficients, as described in Sec. 2.2, to correct for Bias_ft_ (see Methods 4).

**Fig 5:**
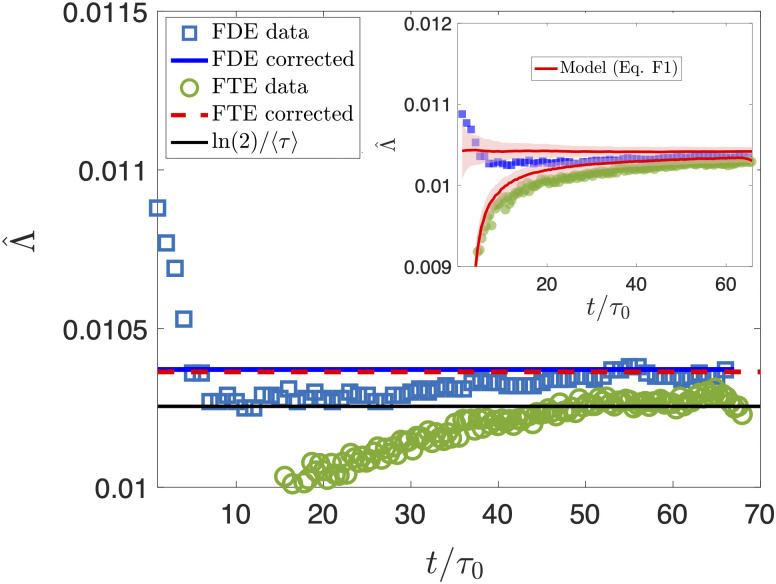
Estimates of Λ obtained using FDE and FTE, including finite-size corrections, applied to data from Ref. [23]. The symbols represent the uncorrected FDE (blue squares) and FTE (green circles). The red dotted line and blue solid line correspond to the finite-time corrected population growth rates for the FTE and FDE, respectively, which are both approximately 0.0104 min^−1^. In the inset, we show that the cell-size control model described in Eqs. (54) and (55), which was fit to the data correlations, quantitatively reproduces the transient behavior observed in both methods. The symbols are the same data as FDE (blue squares) and FTE data (green circles). The solid red lines represent the mean of the estimators over 100 realizations, while the shaded regions denote their standard deviation.

In Fig 5, we show the performance of both FTE and the FDE on the data. Both methods asymptotically converge from below. The best fit lines for both methods are shown in Fig 7. When the two methods are fit and their finite-time error subtracted, the long-time growth rate estimates agree as shown in the red dotted (FTE) and blue (FDE) lines in Fig 5. The long-time population growth rate estimate for the FDE is approximately $1.04\times 10^{-2}\pm 1.97\times 10^{-5}\;{\text{min}}^{-1}$ for the FTE and $1.04\times 10^{-2}\pm 3.11\times 10^{-5}\;{\text{min}}^{-1}$ for the FTE, which are both distinct from the naive estimate, $\ln(2)/\tau_{0}\approx 1.025\times 10^{-2}\;{\text{min}}^{-1}$ (see Fig 7 for the fits). Since the two estimates agree, we have some confidence that the true population growth rate is near this value. Additionally, the finite time coefficient for the FTE (*A* = −0.0060) is about four times larger than FDE (*B* = −0.0016), in agreement with our theory in Sec. 2.2.

Our analysis shows that the method enables inference of relative fitness differences as small as 10^−4^. This level of precision is biologically significant, as even small differences in growth rate can lead to substantial divergence in population sizes over time. To date, there has been no reliable way to infer such small growth rate differences from lineage data. Applying these methods naively leads to a finite-time bias that is on the order of 10^−2^. In contrast, the nonlinear averaging bias behaves more like an all-or-nothing effect: it can be entirely avoided if a sufficient number of lineages are included for a given observation time. One can reduce the finite-time error while avoiding the nonlinear averaging bias by exponentially increasing the number of lineages in the ensemble in tandem with the length of each lineage.

Most of our analysis has focused on understanding the convergence of model-free estimators of the growth rate. However, these estimators of the growth rate can also be used to reverse-engineer a model for the dynamics, albeit with a reconstruction that might not be unique. In the inset of Fig 5 we show that a two-component model for the generation time and log size dynamics that is fit to the correlations of the data quantitatively reproduces the convergence of both methods (see Methods 4.4 for model details).

## 3. Discussion

We have investigated the scaling behavior of errors in two estimators of population growth rate derived from lineage statistics. This problem is very similar to the estimation of free energy differences using Jarzynski’s Equality [31,32] and to the methods for controlling buffer size in ATM networks [34] (see the SI Text, Connection to other work [21]). These problems require sampling rare events, leading to a breakdown of traditional approaches to quantifying the sample distribution. Instead, it is essential to carefully account for extreme value statistics.

An effect of extremal statistics is the introduction of systematic error. Unlike the usual errors in statistical estimators, which stem from the lack of flexibility in the underlying model, this error arises because of poor sampling of the tails. In the context of growth rate estimations, there is an added complication which is the finite-time bias. We have demonstrated that there is, in fact, a trade-off between two types of bias: short-lineage lengths introduce a finite-time bias, while long-lineage lengths result in what we term a nonlinear averaging bias. This latter bias becomes significant when a few lineages dominate the sample averages $\langle \cdot \rangle_{M}$.

From the bias-variance decomposition of the total error, we find that at short times the finite-length bias and the variance between realizations dominate the total error. At long times, there is a phase transition in which the linearization bias dominates. While the finite-length bias can be mitigated through finite-timescaling, addressing the nonlinear averaging bias requires careful selection of lineage durations. By drawing a connection to the REM, we estimate the critical value of $\alpha^{2}=n\ln(2)/\ln M$ for the FTE and $\alpha^{2}=t\ln(2)/\ln M$ for the FTE at which the nonlinear averaging bias becomes dominant. This insight could be valuable in designing experiments that map single-cell data to population growth rates and has broader applications in contexts where one is interested in estimating large deviations of a counting process and its first passage time.

Another intriguing question is how accurately one can infer fitness using lineage-based methods. In principle, these methods can exactly recover the population growth rate from mother machine data. However, their practical accuracy is limited by noise in the data, which constrains the ability to reliably fit the finite-time coefficients. Moreover, perfectly fitting a line—even in principle—requires an infinite amount of data. Our analysis also depends on the location of the critical value $\alpha_{c}$, which is derived from a discrete-time Gaussian model. It is possible that non-Gaussian noise could lead to a different critical α, or even to the absence of a well-defined threshold. Nevertheless, cell-size control models—such as those studied in this paper—have been shown to accurately capture replication dynamics, making this latter concern unlikely to pose a significant issue.

These results suggest a simple, practical protocol for applying lineage-based growth-rate estimators to finite datasets. To reliably infer population growth rates from lineage data, proceed as follows. *First*, determine the maximum usable lineage length before extremal statistics dominate by computing the critical duration *n*_*c*_ (or *t*_*c*_) from Eqs. (21) and (22), which depends on the number of independent lineages *M* and the variability of generation times. *Second*, partition the data so that lineage segments have lengths just below this critical value, ensuring that no small number of exceptionally fast-growing lineages dominates the estimator. *Third*, below the critical point, the finite time bias will dominate. Within this regime, compute the growth-rate estimator as a function of lineage length and verify that it increases approximately linearly when plotted versus the inverse duration (e.g., 1/*n* or 1/*t*). *Fourth*, fit this monotonic region to the expected inverse-leng*t*h scaling: the slope gives the finite-duration bias coefficient (Eq. (13) or Eq. (14)), while the intercept yields the long-time population growth rate. When applied in this order, both fixed-time and fixed-division estimators converge to the same long-term growth rate, yielding robust and reproducible fitness estimates from finite single-cell datasets.

Although our focus is on inferring population growth rates from single-cell lineage data, it is important to note that bulk (population-level) growth-rate inference is itself nontrivial and subject to systematic uncertainties. In batch culture experiments, microbial populations typically exhibit a lag phase followed by exponential growth and eventual deviations from exponential behavior as resources become limiting and carrying capacity is approached [39,40]. Bulk growth rates are commonly inferred by fitting optical density, biomass, or colony-forming unit measurements to phenomenological growth models, such as exponential, logistic, or Gompertz forms, often using only a subset of the growth curve presumed to represent balanced growth [41]. However, the inferred growth rate depends sensitively on how the lag phase is treated, on deviations from pure exponential growth, and on the choice of fitting window and model [42]. Moreover, environmental shifts, diauxic transitions, and physiological heterogeneity can bias bulk estimates, complicating their interpretation as intrinsic fitness measures [43]. Acknowledging these limitations provides a more balanced comparison. Lineage-based approaches introduce systematic biases associated with rare-event sampling, but these biases can be explicitly identified and corrected within the inference framework developed here. In contrast, several dominant sources of error in bulk growth-curve analysis—such as lag-phase treatment and deviations from balanced exponential growth—do not admit a comparably systematic or model-independent correction.

In addition, methods exist to infer how different genotypes affect population growth. Single-variant fitness effects are often estimated in bulk by competing many genotypes in pooled batch culture and tracking their relative abundances over time, frequently using DNA barcodes and deep sequencing so that each genotype’s log-frequency trajectory is approximately linear in time, with slope set by its Malthusian growth-rate difference relative to a reference [44–46]. While powerful and scalable, these assays inherit the same growth-regime ambiguities as standard bulk curves (lag, non-exponential phases, density dependence) and add assay-specific artifacts from serial-dilution bottlenecks, sampling noise, and batch effects across replicates. In barcoded designs, a further dominant failure mode is systematic barcode processing bias: barcode sequence and sample-specific PCR/sequencing conditions can distort read-count trajectories and therefore misestimate slopes, even when the underlying population dynamics are otherwise well behaved [46]. Recent correction methods (e.g., REBAR) can infer and remove substantial barcode-induced bias post hoc, but they do not eliminate the more fundamental ambiguities tied to growth-phase selection and history dependence in bulk cultures. In this sense, lineage-based inference offers a complementary route to variant fitness that connects directly to single-cell statistics, while making a different—and in our setting, explicitly correctable—set of finite-data assumptions.

Regarding the connection to of lineage-based methods REM, it is intriguing to explore more rigorously whether the limiting behavior of $\hat{\Lambda }$ can be understood using similar techniques. It is well-known (see, e.g., [47,48]) that the REM exhibits two phase transitions at $\beta_{1}=\sqrt{\ln2}$ and $\beta_{2}=2\sqrt{\ln2}$. For $\beta >\beta_{2}$, the partition function *Z*_*N*_ fails to obey the Law of Large Numbers, meaning that $Z_{N}/E[Z_{N}]$ no longer converges to one in probability. For $\beta >\beta_{1}$, the Central Limit Theorem no longer holds, implying that $\left(Z_{N}-E[Z_{N}])/\text{var}(Z_{N}\right)$ does not exhibit Gaussian fluctuations. A deeper understanding of these phase transition behaviors in $\hat{\Lambda }$ could enable more accurate inference in the future.

Lastly, understanding the biases and their trade-offs is important for applications involving Jarzynski’s Equality and ATM networks (see the SI Text, Connection to other work [21]). More broadly, such trade-offs arise in thermodynamic inference problems, including the estimation of entropy and entropy production rate (EPR) [49–52]. One widely used approach is the *plug-in* method, where empirical probabilities estimated from time-series data are directly substituted into information-theoretic expressions—in this case, the Kullback–Leibler divergence between forward and time-reversed trajectories. In Ref. [51], this method was shown to suffer from the same systematic errors highlighted here: finite-time biases arising from splitting trajectories into blocks of limited duration, and finite-sample biases due to limited statistics. However, unlike in the present work, no phase transition was observed. Also, there are some EPR methods that involve waiting time distributions [53,54], which have similarities to generation times. A precise understanding of biases inherent to finite data will allow for accurate inference of fundamental properties.

In addition, similar biases arise in importance sampling methods used to compute large deviation functions, such as cloning [55–58]. In this method, many independent copies (clones) of the system are simulated in parallel. By the law of large numbers, some clones naturally exhibit the desired rare behavior. The algorithm then amplifies these trajectories by duplicating the clones that realize the rare dynamics, while deleting those that do not. The cloning algorithm has a finite time bias as well as a finite clone bias, which is analogous to the finite lineage bias in our work.

## 4. Methods

### 4.1. Relation of growth rate estimators to large deviation theory

We consider the general setting in which single-cell generation times evolve stochastically according to some process $\left\{\tau_{k}\right\}$. This need not be a Markov process, but to be concrete we imagine there is some underlying phenotype (e.g., gene expression) $x_{k}\in R^{d}$ which evolves according to a Markov process with transition operator $h(x_{k+1}|x_{k})$, and that generation times are deterministic functions of the phenotype τ(x). This modeling framework can capture all existing models of single-cell dynamics [59–61]. We let $T_{n}=\sum_{k=1}^{n}\tau_{k}$ denote the time at which the *n*th cell in a lineage divides.

The long-term growth rate is related to the lineage-to-lineage fluctuations in the counting process,


$$\begin{array}{c} N_{t}=\max\left\{n:\sum_{i=1}^{n}\tau_{i}<t\right\}\;, \end{array}$$ (23)

and Λ is given by [25]


$$\begin{array}{c} \Lambda =\psi (\ln(2)) \end{array}$$ (24)

where ψ(z) is the scaled-cumulant generating function,


$$\begin{array}{c} \psi (z)=\lim_{t\to \infty }\frac{1}{t}\ln E[e^{N_{t}z}]\;. \end{array}$$ (25)

Here, *z* is the conjugate variable to *N*_*t*_, and E[·] represents the expected value with respect to the lineage distribution within the *fixed-time ensemble (FTE)*, as illustrated in Figs 1B and C. The intuition behind Eq. (24) is that lineages with *n* divisions on average contribute $2^{N_{t}}$ cells to the final population, hence the total population size is on average $E[2^{N_{t}}]\sim e^{\Lambda t}$.

Given lineage samples $\left\{N_{t}^{\left(i\right)}\right\}$ which come from repeated experiments or splitting a long lineage into blocks, ψ(z) and hence Λ can in principle be estimated by replacing the expectation with an empirical average:


$$\begin{array}{c} {\hat{\psi }}_{t}(z)=\frac{1}{t}\ln{\left\langle e^{N_{t}^{\left(i\right)}z}\right\rangle }_{M} \end{array}$$ (26)

where $\langle \cdot \rangle_{M}$ is the empirical average over *M* samples. An estimator of Λ is then ${\hat{\Lambda }}_{1}(t,M)={\hat{\psi }}_{t}(\ln(2))$ which is equivalent to Eq. (4), and is always a biased estimator.

These formulas naturally connect to the large deviations of *N*_*t*_ through the Gärtner-Ellis Theorem, which states that


$$\begin{array}{c} -\lim_{t\to \infty }\frac{1}{t}\ln P(N_{t}/t=x)=I(x), \end{array}$$ (27)

where *I*(*x*) is the *large deviation rate function*, defined as the Legendre-Fenchel transform $\psi^{*}(x)$ of ψ(x). Informally, Eq. 27 tells us that the fluctuations in $N_{t}/t$ decay exponentially with time: $P(N_{t}/t=x)\approx A_{t}e^{-tI(x)}$, where *A*_*t*_ is the normalization constant.

Equation 27 states a large deviation principle, which we assume in order to apply large deviation theory to infer the population growth rate. Specifically, we assume that for the FDE the sequence of division times $\tau_{k}$ (or, equivalently for the FTE, the cumulative number of divisions up to time t) obeys a large deviation principle. This condition is typically met when the $\tau_{k}$ (or division counts) are only finitely correlated, ensuring the law of large numbers applies, and when the observation time $T_{n}=\sum_{k=1}^{n}\tau_{k}$ (or equivalently the number of divisions n) is much longer than the correlation time, i.e., n≫1.

In Ref. [25], it was shown that due to the large deviation structure, the estimator ${\hat{\Lambda }}_{1}$ exhibits a somewhat surprising non-monotonic convergence. This phenomenon is related to the so-called linearization effect [33], which can be understood as follows. The integral $E[e^{xz}]\approx \int p_{t}(x)e^{xzt}\;dx$ is dominated by a value $x^{*}=N_{t}^{*}/t$ for which $\psi (z)=x^{*}z-I(x^{*})$ is extremal. Therefore, to obtain an accurate estimate of $E[e^{N_{t}z}]$ from finite samples, we must have a high probability of sampling $N_{t}=N_{t}^{*}$. However, when *t* is large, this is an exponentially rare event, requiring an exponentially large number of samples.

As shown in Ref. [26], the growth rate can alternatively be expressed in terms of the scaled cumulant generating function (SCGF) of *T*_*n*_, $\phi (z)=\lim_{n\to \infty }n^{-1}\ln E[e^{T_{n}z}]$, as


$$\begin{array}{c} -\phi (-\Lambda )=\ln(2). \end{array}$$ (28)

This result arises from the fact that $\phi (z)=\psi^{-1}(z)$ for the SCGFs of a counting process and its dual first-passage time process [62], which we refer to as the *fixed-divisions ensemble (FDE)* (Note that in Ref. [26], this estimator was called the Generalized Euler-Lotka (GEL) Equation). A comparison of these ensembles is shown in Figs 1B and C. Additionally, the large deviation rate function for $T_{n}/n$, denoted by *J*, can be related to *I* through the expression *J*(*y*) = *xI*(1/*x*) [62].

Following Ref. [26] and Eq. (5), an estimator ${\hat{\Lambda }}_{2}$ can be obtained as the positive solution to the nonlinear equation


$$\begin{array}{c} -{\hat{\phi }}_{n}(-{\hat{\Lambda }}_{2}(n,M))=\ln(2), \end{array}$$ (29)

where ${\hat{\phi }}_{n}(z)$ is the empirical SCGF for the dual process:


$$\begin{array}{c} {\hat{\phi }}_{n}(z)=\frac{1}{n}\ln{\left\langle e^{T_{n}^{\left(i\right)}z}\right\rangle }_{M}. \end{array}$$ (30)

Since ${\hat{\phi }}_{n}(z)$ is convex, ${\hat{\Lambda }}_{2}(n,M)$ is uniquely defined by Eq. (29).

Our primary goal is to analyze the convergence behavior of the FTE and FDE ensembles, denoted by ${\hat{\Lambda }}_{1}$ and ${\hat{\Lambda }}_{2}$, respectively, as a function of the parameters *M* and *t* (for the FTE) and *M* and *n* (for the FDE). Although the dual estimator ${\hat{\Lambda }}_{2}$ also appears to have systematic errors from the linearization effect, it is unclear whether one estimator consistently outperforms the other or how their convergence patterns are influenced by specific model details.

In this work, we demonstrate that a similar result can be obtained for the FDE estimator as follows: We can express the average on the left-hand side of Eq. (29) in terms of the large deviation function (see the SI 4.1),


$$\begin{array}{c} \left\langle e^{-T_{n}^{\left(i\right)}{\hat{\Lambda }}_{2}}\right\rangle =\frac{K_{n}}{n}\int p(t)e^{-nt{\hat{\Lambda }}_{2}}\;dt\;, \end{array}$$ (31)

where *t* = *T*/*n* and *K*_*n*_ is a normalization constant.

### 4.2 Derivation of the population growth rate of the simulation model SCE

If the large deviation rate function is quadratic (e.g., *T*_*n*_ is Gaussian), then we can do a cumulant expansion of Eq. (30) and truncate at second order. Starting with the series expansion


$$\begin{array}{c} \ln E[e^{T_{n}z}]=\sum_{k=1}^{\infty }\kappa_{k}\frac{z^{k}}{k!}, \end{array}$$ (32)

and dropping all but the first two terms, and substituting into Eq. (28) yields


$$\begin{array}{c} \tau_{0}\Lambda -\frac{\sigma_{T}^{2}}{2}\Lambda^{2}=\ln(2) \end{array}$$ (33)

where


$$\begin{array}{c} \tau_{0}=\lim_{n\to \infty }\frac{E[T_{n}]}{n} \end{array}$$ (34)

$$\begin{array}{c} \sigma_{T}^{2}=\lim_{n\to \infty }\frac{var(T_{n})}{n}. \end{array}$$ (35)

Solving the quadratic equation for Λ yields Eq. (11). Since this formula is exact when the large deviation rate function is quadratic, and thus the statistics of *T*_*n*_ are Gaussian, we call it the *Second Cumulant Expansion (SCE)*. However, for real data, the accuracy of this approximation is not known a priori.

To illustrate the limitations of the SCE, we present a counterexample where the method does not work. We examine a model in which generations are independent and $\tau =t_{0}$ with probability 1/2, and τ=1 otherwise. Because this is an independent generation time model, the tree and lineage distributions are identical, resulting in the same equation from both the Euler-Lotka and FDE:


$$\begin{array}{c} e^{t_{0}\Lambda }=e^{(t_{0}-1)\Lambda }+1\; \end{array}$$ (36)

which can be numerically solved for Λ.

Now, we can determine the growth rate for the SCE by substituting


$$\begin{array}{c} \tau_{0}=\frac{t_{0}+1}{2} \end{array}$$ (37)

and


$$\begin{array}{c} \sigma^{2}=\frac{(t_{0}-1{)}^{2}}{4} \end{array}$$ (38)

into Eq. (11), yielding


$$\begin{array}{c} \Lambda =\frac{\frac{4\ln(2)}{t_{0}+1}}{1+\sqrt{1-2\ln(2){\left(\frac{t_{0}-1}{t_{0}+1}\right)}^{2}}}. \end{array}$$ (39)

Note that the SCE simplifies to the naïve estimate $\ln(2)/\tau_{0}$ when *t*_0_ = 1, reflecting the zero-noise limit.

**Fig 6:**
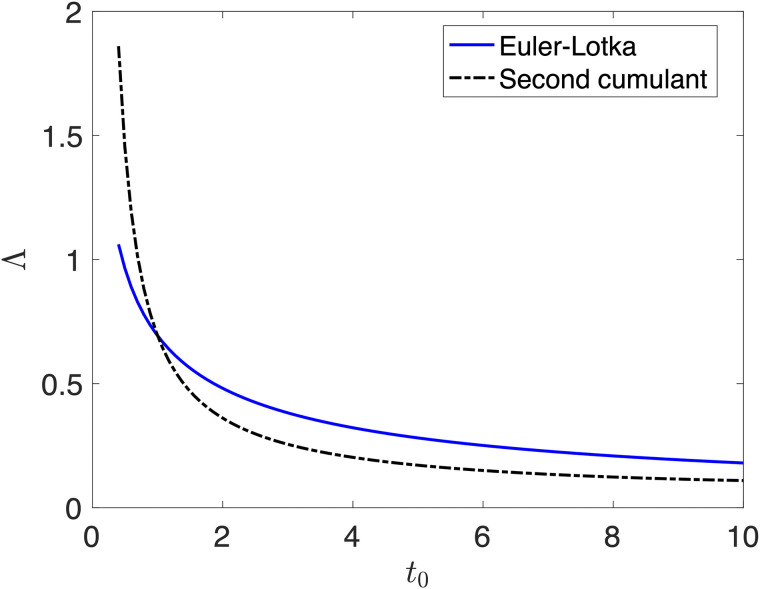
The figure shows an illustrative example where the SCE approximation fails. In this example, generation times are uncorrelated, and chosen to be $\tau =t_{0}$ with probability 1/2 and τ=1 otherwise. The (exact) result of the Euler-Lotka equation is given by the solid blue line (Eq. (36)), and is compared with the SCE method (black, dashed line), which relies only on the first and second cumulants of the distribution (Eq. (39)).

The predictions of Eqs. (36) and (39) are compared in Fig 6, showing that the second cumulant method is inaccurate. Indeed, even for the case of independent generation times, the (exact) Euler-Lotka equation tells us that knowledge of the entire generation time distribution is needed; hence, the mean and variance are insufficient - therefore, the SCE cannot be guaranteed to yield accurate results for non-Gaussian generation time distributions.

**Fig 7:**
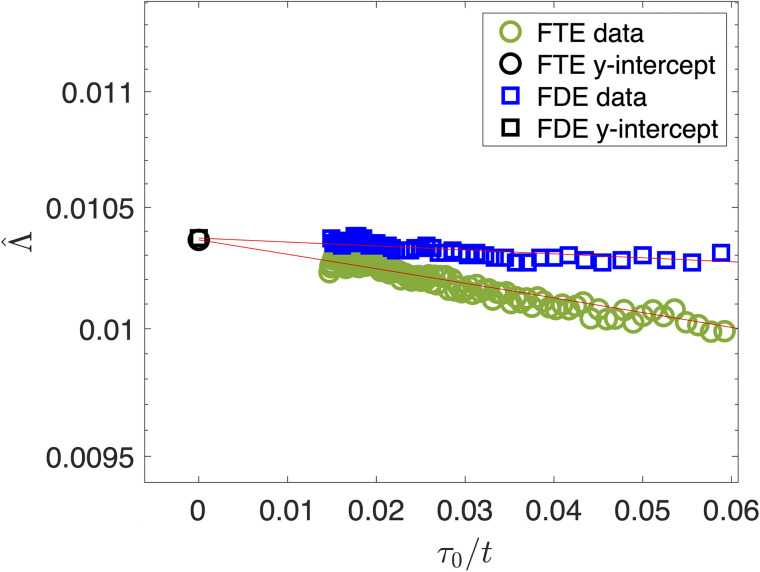
Estimates of Λ using the FDE and FTE with finite size corrections with the same data as Fig. 5. By plotting as a function of $\tau_{0}/t$, we can fit the convergence to a line. The symbols represent the FDE (blue squares) and FTE (green circles) applied with no corrections. The solid red lines represent the fit and the black symbols are y-intercepts which give the long-time population growth rate.

### 4.3. The mapping of FDE and FTE to REM

We will first show the connection of the FDE with REM. We focus on the asymptotic behavior of the free energy for REM density, defined as


$$\begin{array}{c} f_{N}(\beta )=-\frac{1}{\beta N}\ln Z_{N}, \end{array}$$ (40)

with the limit of the quenched average given by $\bar{f}(\beta )=\lim_{N\to \infty }E[f_{N}(\beta )]$. In the thermodynamic limit, we find the free energy to be [38]:


$$\begin{array}{c} \bar{f}(\beta )=\left\{ \begin{array}{cc} -\frac{\beta }{4}-\frac{\ln2}{\beta }, & \text{if }\beta \leq \beta_{c},\\ -\sqrt{\ln2}, & \text{if }\beta >\beta_{c}. \end{array}\right. \end{array}$$ (41)

where $\beta_{c}=2\sqrt{\ln2}$ is the critical inverse temperature. The free energy in the high temperature regime $\beta <\beta_{c}$ is entropically dominated and can be obtained by replacing *Z*_*N*_ with $E[Z_{N}]$ in Eq. (40), avoiding the need to evaluate the quenched average. This is due to a concentration of the Gibbs measure, which is well established for the REM. In contrast, for the low temperature regime $\beta \geq \beta_{c}$ the free energy density is determined by the extremal energy levels. It can be shown that the transition corresponds to the breakdown of the Law of Large Numbers for *Z*_*N*_ [48]. Within this phase, the partition function is dominated by a few energy levels. As we show below, within our context the growth rate estimators are dominated by a few extremal lineages past the phase transition.

For the FTE Eq. (30) with the exponent shown in Eq. (17) can be rewritten in terms of the (finite-size) free energy density of the REM by introducing the temperature parameter $\beta_{FDE}(z)=z\sigma \alpha_{FDE}$:


$$\begin{array}{c} \begin{array}{cc} \lim_{n\to \infty }E[{\hat{\phi }}_{n}(-z)]= & \;\;\;\;-\frac{\beta_{FDE}(z)}{\alpha_{FDE}^{2}}f_{N}(\beta_{FDE}(z))\\ & \;\;\;\;-\frac{\ln(2)}{\alpha_{FDE}^{2}}-z\tau_{0}. \end{array} \end{array}$$ (42)

Equation (42) is an exact equation but we can’t solve for $\hat{\Lambda }$ analytically, since we don’t have a closed formula for the *finite-size* free energy density $f_{N}(\beta (z))$. However, we can study the large *N* behavior by replacing $f_{N}(\beta (z))$ with the formula for $\bar{f}(\beta (z))$ given in Eq. (41). The resulting equation can be solved to yield an estimate of the FDE estimator $E[\hat{\Lambda }]\approx {\hat{\Lambda }}_{REM}(\alpha_{FDE})$ which has the explicit formula of Eq. (19)


$$\begin{array}{c} {\hat{\Lambda }}_{REM}(\alpha )=\left\{ \begin{array}{cc} \Lambda & \alpha <\alpha_{c}\\ \frac{2\ln(2)(1-\alpha^{2}/2)}{\tau_{0}(2\alpha \sigma /\tau_{0}\sqrt{\ln(2)}-\alpha^{2})} & \alpha \geq \alpha_{c} \end{array}\right. \end{array}$$ (43)

with the critical value of α given by


$$\begin{array}{c} \alpha_{c}=\frac{2\sqrt{\ln(2)}}{\Lambda \sigma }\;. \end{array}$$ (44)

Here Λ is given by Eq. (11). Note that a useful approximation to $\alpha_{c}$ is


$$\begin{array}{c} \alpha_{c}\approx 2/(\sqrt{\ln(2)}{CV}_{T})\;. \end{array}$$ (45)

However, it is important to note that our definition of Bias_nl_ is based on $E[\hat{\Lambda }]$, where the expectation is taken after solving Eq. (30). In contrast, to derive Eq. (43), we employed Eq. (41), where the expectation is taken before solving Eq. (42). Namely, the ordering of limits is different which could lead to systematic differences. Most likely, the deviations from the exact value seen in Fig 4 are due to finite-size effects. For a given α, both *n* and ln(M) should go to infinity while leaving the ratio n/lnM fixed. Deviations from REM were also seen in sampling Jarzynski’s Equality [31].

In the FTE, we can similarly make the connection to the REM by viewing $\ln(2)N_{t}^{\left(i\right)}$ as the scaled energy levels. Since *N*_*t*_ is a counting variable, Eq. (41) is no longer valid. The REM with discrete energy levels has been studied in Refs. [63–65], where both binomial and Poisson energy distributions have been studied. We found that a Gaussian approximation nevertheless seems to capture the convergence very well in the regimes we are interested in.

In our earlier work [25], we derived the rate function for the autoregressive generation time model:


$$\begin{array}{c} I(y)=\frac{(1-c{)}^{2}}{2\sigma_{\tau }^{2}}(\tau_{0}-1/y{)}^{2}. \end{array}$$ (46)

A Taylor expansion around $y=1/\tau_{0}$ yields a Gaussian approximation:


$$\begin{array}{c} \ln(2)N_{t}\approx t\ln(2)/\tau_{0}+\ln(2)\sqrt{t}\sigma_{y}X_{i}. \end{array}$$ (47)

This motivates the following definitions:


$$\begin{array}{c} \alpha_{FTE}=\sqrt{\frac{2t}{N}}, \end{array}$$ (48)

and


$$\begin{array}{c} \beta_{FTE}=\ln(2)\sigma_{y}\alpha_{FTE}. \end{array}$$ (49)

where $\sigma_{y}=\sigma_{\tau }^{2}/(1-c{)}^{2}$. Similar to the approach taken for the FDE, we can rewrite Eq. (26) in terms of $f_{N}(\beta )$:


$$\begin{array}{c} {\tilde{\Lambda }}_{REM}(\alpha )=\frac{\ln(2)}{\tau_{0}}+\frac{1}{\alpha^{2}}\left(-\beta f_{N}(\beta )-\ln(2)\right). \end{array}$$ (50)

Once we replace $f_{N}(\beta )$ with ${\bar{f}}_{N}(\beta )$ we obtain


$$\begin{array}{c} {\tilde{\Lambda }}_{REM}(\alpha )=\left\{ \begin{array}{cc} \tilde{\Lambda } & \alpha <\alpha_{c}\\ \frac{\ln(2)}{\tau_{0}}+\frac{1}{\alpha^{2}}(\sqrt{\ln(2)}-\ln(2)) & \alpha \geq \alpha_{c} \end{array}\right. \end{array}$$ (51)

where $\tilde{\Lambda }=\ln(2)/\tau_{0}+\ln(2{)}^{2}\sigma_{y}^{2}/4$, which is obtained by Taylor expanding Λ, given by Eq. 11, in powers of $\sigma_{y}^{2}$. Note that the transition occurs at the same critical α for both estimators but the decay to the naive solution of $\ln(2)/\tau_{0}$ is different.

In Fig 2 and in Ref. [25], we used σ=0.2. For this value of the noise, $\alpha_{c}\approx 12$, and *n* < 300 is needed to avoid the linearization effect. This analysis qualitatively agrees with a more heuristic approach in Ref. [25] where we obtained the criterion $n<\frac{\ln(M)}{\sigma^{2}\ln(2)}\approx 100$ divisions.

### 4.4. Fits of the experimental data

Simulated data was generated by fitting autoregressive models to the experimental *E. coli* data. First, we fit the autoregressive model given by Eq. (10). This was achieved by performing a simple linear regression. Next, in order to account for the fact that cell size is regulated, we fit a multivariate autoregressive model, where log cell length was included as a predictor. This takes the form


$$\begin{array}{c} x_{i+1}=Ax_{i}+b+\xi_{i} \end{array}$$ (54)

Here, $A\in R^{2\times 2}$ and $b\in R^{2}$ are coefficients to be fitted and $\xi_{i}\in R^{2}$ is a noise vector. The regression variable is


$$\begin{array}{c} x_{i}=\left[\begin{array}{c} \ln s_{i}\\ \tau_{i} \end{array}\right] \end{array}$$ (55)

and *s*_*i*_ is the size of the *i*th cell at birth.

We used standard least squares for regression with multiple response variables [28] to fit the coefficients *A* and **b**, as well as the noise magnitudes.

Note that by simply including the additional variable of log cell-size, cell size is automatically regulated. We could have alternatively included growth rates, instead of generation time and/or log fold change in size as predictors. This was the approach taken in [66]. However, we found that for the purpose of predicting growth rate and the convergence pattern of the FTE and FDE, simply adding the additional predictor of size was sufficient.

## Supporting information

S1 Text Supplementary methods and derivations for “Extremal events dictate population growth rate inference.” Provides detailed model definitions and analytical results: (i) Cell-size control model used in simulations; (ii) Transport (von Foerster) equation derivation of the FDE estimator and discussion of finite-time bias; (iii) Connections to related work, including Jarzynski’s Equality–based estimators and admission control in ATM networks; (iv) Derivation of the finite-time growth rate for the FTE under Gaussian/AR(1) lineage statistics. Includes full equations, assumptions, and references [67].
(PDF)
