# Supplementary material for: Extremal events dictate population growth rate inference
Source: PLoS Comput Biol. 2026 May 13;22(5):e1014088. doi: 10.1371/journal.pcbi.1014088 (PMC13421774; doi:10.1371/journal.pcbi.1014088)
Supplement: S1 Text — Provides detailed model definitions and analytical results: (i) Cell-size control model used in simulations; (ii) Transport (von Foerster) equation derivation of the FDE estimator and discussion of finite-time bias; (iii) Connections to related work, including Jarzynski’s Equality–based estimators and admission control in ATM networks; (iv) Derivation of the finite-time growth rate for the FTE under Gaussian/AR(1) lineage statistics. Includes full equations, assumptions, and references [67]. (PDF) [file pcbi.1014088.s001.pdf]

Supplemental Information for Extremal events dictate population growth rate inference

Trevor GrandPre[1-3]◑, Ethan Levien[4]◑, Ariel Amir[5]*

1 Department of Physics, Washington University in St. Louis, St. Louis, MO, USA
2 National Institute for Theory and Mathematics in Biology, Northwestern University and The University of Chicago, Chicago, IL, USA
3 Department of Physics, Princeton University, Princeton, NJ, USA
4 Department of Mathematics, Dartmouth College, Hanover, NH, USA
5 Department of Physics of Complex Systems, Weizmann Institute of Science, Rehovot, Israel

◑These authors contributed equally to this work.
* ariel.amir@weizmann.ac.il

A Cell-size control model

In Fig. 1 of main text, we show simulations of a biophysically grounded model of microbial growth, commonly used to explore mechanisms of cellular size homeostasis [1, 2]

This model presumes that individual cells grow exponentially until they reach a division size $v_{\rm d}$, which is a function of their size at birth, $v_{\rm b}$. For exponentially growing cells, the generation time τ follows $v_{\rm d} = v_{\rm b} e^{\lambda\tau}$, or equivalently,

$$\tau = \frac{1}{\lambda} \ln\left(\frac{v_{\rm d}}{v_{\rm b}}\right), \tag{S1}$$

where λ represents the growth rate at the single-cell level. Assuming symmetric cell division, the size at birth, $v_{\rm b}$, is half the size of the mother cell at division. To incorporate phenotypic variability, the model includes random fluctuations in both growth rates and division volumes. Specifically, each cell's growth rate λ and division volume $v_{\rm div}$ are modeled by

$$\ln\lambda = \ln\lambda_0 + \eta_\lambda, \tag{S2}$$

$$v_{\rm div} = 2(1-\alpha)v_{\rm birth} + 2\alpha v_0 + \eta_v, \tag{S3}$$

where η_λ and η_v are independent normal random variables with variances σ_λ^2 and σ_v^2, respectively [3]. The growth rates follow a log-normal distribution with small noise, and the division timing is based on cell volume. Here, α dictates the cell-size regulation approach: for $\alpha = 1$ (a "sizer" strategy), cells divide at a target size, whereas for $\alpha = 1/2$ (an "adder" strategy), cells increase by a consistent size v_0 from birth to division.

B Transport equation derivation of FDE

We now present an alternative derivation of the FDE estimator using the von Foerster equation approach. This method has the advantage of clarifying the numerical results.

In essence, the significantly smaller bias of the FDE estimator arises from the absence of finite-time bias, provided that generation times are uncorrelated across generations ($c = 0$ within the model shown in Eq. 10 of the main text).

To understand this more precisely, recall that we assume the Markov transition operator for the generation time dynamics, $h(x|y)$, is such that x_k converges to a unique stationary distribution $\rho(x)$ for any initial x_0 [4]. Given that $N(a,x,t)$ represents the number of cells with age a and phenotype x at time t, the von Foerster equation is expressed as

$$\frac{\partial}{\partial t}N(a,x,t) = -\frac{\partial}{\partial a}N(a,x,t), \quad a < \tau(x) \tag{S4}$$

$$N(0,x,t) = 2\int_0^\infty h(x|x')N(\tau(x'),x',t)\,dx'. \tag{S5}$$

(This form is slightly different from most standard definitions, where the phenotype x is not considered.) The total number of cells at time t is given by $N(t) = \int_0^\infty \int_0^a N(a,x,t)\,da\,dx$, and given our assumptions on x, for large t, $\phi(a,x,t) = N(a,x,t)/N(t)$ converges to a steady-state $\varrho(x,\tau)$ that satisfies

$$-\frac{\partial}{\partial a}\varrho(a,x) = -\Lambda\varrho(a,x) \implies \varrho(a,x) = \varrho(0,x)e^{-\tau(x)\Lambda}\ . \tag{S6}$$

This leads to the equation

$$\begin{aligned}\varrho(0,x) &= 2\int h(x|x')\varrho(x')\,dx' \\ &= 2\int h(x|x')\varrho(0,x')e^{-\tau(x')\Lambda}\,dx'\ .\end{aligned} \tag{S7}$$

Integrating over x:

$$1 = 2\int \varrho_b(x)e^{-\tau(x)\Lambda}\,dx \tag{S8}$$

where $\varrho_b(x)$ is the "birth distribution" of x. Equation (S8) is a generalization of the well-known Euler-Lotka equation.

Note that in Eq. (S8), $\varrho_b(x)$ must be obtained from the population distribution. To derive a relation that depends solely on lineage statistics, we iteratively replace $\varrho(0,x')$ using Eq. (S7), yielding

$$\begin{aligned}\varrho_b(x_n) &= 2^n \int\cdots\int \varrho_b(x_0)\prod_{i=1}^n h(x_i|x_{i-1})e^{-\tau(x_{i-1})\Lambda}\,dx_i\,dx_0 \\ &= 2^n \int\cdots\int \varrho_b(x_0)e^{-T_n\Lambda}\prod_{i=1}^n h(x_i|x_{i-1})\,dx_i\,dx_0\ ,\end{aligned} \tag{S9}$$

where $T_n = \sum_{i=1}^n \tau(x_i)$ is the total time along a lineage of n divisions.

Integrating over x_n, taking logarithms, and dividing by n gives

$$-\ln 2 = \frac{1}{n}\ln \mathbb{E}_{T,\varrho_b}\left[e^{-T_n\Lambda}\right], \tag{S10}$$

where $\mathbb{E}_{T,\varrho_b}$ denotes the expectation of T_n, with the first cell drawn from ϱ_b.

Equation (S10) holds for all n, but if we replace ϱ_b with another distribution, $\tilde{\varrho}$, we must consider the limit of a large number of divisions to derive an equivalent form of Eq. (28):

$$-\ln 2 = \lim_{n\to\infty}\frac{1}{n}\ln \mathbb{E}_{T,\tilde{\varrho}}\left[e^{-T\Lambda}\right]. \tag{S11}$$

This derivation is distinct from that in Ref. [5] and has important implications for the estimator in Eq. (29). The finite-time bias arises only from the discrepancy between the initial distribution $\tilde{\varrho}$, assumed to be the lineage distribution, and the population distribution ϱ_b. Consequently, this bias vanishes when there are no correlations (i.e., when $h(x|y) = h(x)$) for FDE. This contrasts with the FTE estimator [6], where there is always a finite-time correction regardless of the initial distribution or correlations between generation times.

C Connection to other work

C.1 Jarzynski's Equality estimators

Here, we discuss in greater detail the connection to Jarzynski's Equality estimators of free energy differences, with particular emphasis on the results presented in Refs. [7, 8]. These studies examine a physical system parameterized by the system size n, which is driven out of equilibrium between two configurations, Γ_1 and Γ_2. The well-known Jarzynski's Equality relates the work performed during this process, W, to the free energy difference between the two configurations, ΔF, through the expression

$$\Delta F = -\frac{1}{\beta} \ln \mathbb{E}[e^{-\beta W}]. \tag{S12}$$

Using Equation S12, the free energy difference can be estimated by substituting the expectation with the empirical average:

$$\widehat{\Delta F} = -\frac{1}{\beta} \ln \left\langle e^{-\beta W^{(i)}} \right\rangle. \tag{S13}$$

The connection to growth rate estimation becomes evident in this context.

In Ref. [7], the authors explain that for small M, $\widehat{\Delta F}$ is a biased estimator of ΔF and analyze this bias by relating the quenched average $\mathbb{E}[\widehat{\Delta F}]$ to the free energy of the REM, obtaining results very similar to ours for the bias.

Interestingly, they also derive finite size corrections, which would correspond to finite time corrections to the finite lineage bias in our case. These come from earlier work on finite size correction to the REM. It appears that these corrections are less relevant in our application, since our data is in the high temperature regime of the REM.

C.2 ATM networks

Estimating large deviation rate functions also plays a significant role in admission control for Asynchronous Transfer Mode (ATM) networks. For a detailed discussion of this application, we refer to Ref. [9]. The basic setup is as follows: The goal is to process an input stream (e.g., service requests) where arrivals occur at stochastic times and are serviced at a constant rate. These requests are queued in a buffer of length b, and once the buffer is full, additional calls are discarded. The objective is to optimize the service rate to minimize or prevent call loss.

In Ref. [9], it is shown that this problem can be reduced to estimating the large deviation rate function I of the process A_t, which counts the arrivals of requests in the queue. Specifically, the tail probability of the queue length Q is given by

$$P(Q > q) \sim e^{-q\delta}, \tag{S14}$$

where

$$\delta = \min\left\{\frac{I(a)}{a} : a \geq 0\right\}. \tag{S15}$$

Thus, δ can be derived from an estimate of the Scaled Cumulant Generating Function (SCGF) of A_t. This connection has motivated rigorous analysis of the convergence properties of SCGF estimators.

To our knowledge, the interplay between the fixed time and fixed count ensembles has not been explored in this context.

D derivation of finite-time growth rate for FDE

We can compute the finite-time population growth rate for FDE by making the approximation that the lineage distribution is multivariate normal, that is,

$$p_{\text{lin}}(\boldsymbol{\tau}) \approx \frac{e^{-(\boldsymbol{\tau}-\bar{\boldsymbol{\tau}})^T K_n^{-1} (\boldsymbol{\tau}-\bar{\boldsymbol{\tau}})}}{(2\pi)^{n/2} \det K_n^{1/2}}. \tag{S16}$$

Here, K_n is the matrix with entries $K_{n,i,j} = \text{cov}(\tau_i, \tau_j)$ and $\bar{\boldsymbol{\tau}} = \mathbb{E}[\boldsymbol{\tau}] = (\bar{\tau}, \cdots, \bar{\tau})^T$. This formula will be exact for any Gaussian process, e.g., any autoregressive process. In particular, for an AR(1) process,

$$\tau_{n+1} = \bar{\tau}(1-c) + c\tau_n + \eta_n \tag{S17}$$

we would have $K_{n,i,j} = \sigma_\eta^2/(1-c^2)c^{|i-j|}$.

Using the well-known formula for the moment generating function of a multivariate normal (see e.g. Ref. [10]),

$$\mathbb{E}\left[e^{-\Lambda \mathbf{1}^T \boldsymbol{\tau}}\right] = e^{-\Lambda \mathbf{1}^T \left(\bar{\boldsymbol{\tau}} - \frac{\Lambda}{2} K_n \mathbf{1}\right)} = e^{-\Lambda n \bar{\tau} + \frac{\Lambda^2}{2} \mathbf{1}^T K_n \mathbf{1}} \tag{S18}$$

In the special case of an AR(1) process with $\sigma_\xi^2 = (1-c^2)\sigma_\tau^2$, we get

$$\frac{\mathbf{1}^T \mathbf{K_n} \mathbf{1}}{n} = -\frac{\left(n\left(c^2-1\right) - 2c\left(c^n-1\right)\right)\sigma_\tau^2}{n(c-1)^2} \tag{S19}$$

where the approximation neglects terms exponentially small in c. Using (S19), for the variance of the FDE estimator, we now have to solve the following equation to solve the FDE equation:

$$\frac{\Lambda^2}{2n} \mathbf{1}^T \mathbf{K_n} \mathbf{1} - \Lambda \tau_0 + \ln(2) = 0 \ . \tag{S20}$$

Solving for Λ and multiplying the top and bottom by

$$\tau_0 + \sqrt{\tau_0^2 + \frac{2(1+c)\sigma_\tau^2 \ln(2)}{1-c}} \tag{S21}$$

gives the n-dependent growth rate to be

$$\Lambda_{\text{n}} = \frac{2\ln(2)/\tau_0}{1 + \sqrt{1 - \frac{\sigma_\tau^2((1-c^2)2\ln(2)n + 4c(c^n-1)\ln(2))}{(1-c)^2 \tau_0^2 n}}} \ . \tag{S22}$$

We can write Eq. S22 in terms of the asymptotic solution as n goes to infinity,

$$\Lambda_{\text{n}} = \Lambda + \frac{B}{n} \ , \tag{S23}$$

where Λ is shown in Eq. (11) of the main text, and

$$\begin{aligned} B =& \frac{4c\ln(2)^2 \sigma_\tau^2 \sqrt{1 - 2\ln(2)\frac{\sigma_\tau^2}{\tau_0^2}\frac{1+c}{1-c}}}{(c-1)\,\tau_0\left((c-1)\,\tau_0^2 + 2\sigma_\tau^2 \ln(2)\,(1+c)\right)} \\ &\times \frac{1}{\left(1 + \sqrt{1 - 2\ln(2)\frac{\sigma_\tau^2}{\tau_0^2}\frac{1+c}{1-c}}\right)}. \end{aligned} \tag{S24}$$

References

1. Ho PY, Lin J, Amir A. *Modeling cell size regulation: From single-cell-level statistics to molecular mechanisms and population-level effects.* Annu Rev Biophys. 2018;47(1):251–271.
2. Amir A. *Cell size regulation in bacteria.* Phys Rev Lett. 2014;112(20):208102.
3. Lin J, Amir A. *From single-cell variability to population growth.* Phys Rev E. 2020;101(1):012401.
4. Levien E, Kondev J, Amir A. *The interplay of phenotypic variability and fitness in finite microbial populations.* J R Soc Interface. 2020;17(166):20190827.
5. Pigolotti S. *Generalized Euler-Lotka equation for correlated cell divisions.* Phys Rev E. 2021;103(6):L060402.
6. Levien E, GrandPre T, Amir A. *Large deviation principle linking lineage statistics to fitness in microbial populations.* Phys Rev Lett. 2020;125(4):048102.
7. Suárez A, Silbey R, Oppenheim I. *Phase transition in the Jarzynski estimator of free energy differences.* Phys Rev E. 2012;85(5):051108.
8. Palassini M, Ritort F. *Improving Free-Energy Estimates from Unidirectional Work Measurements: Theory and Experiment.* Phys Rev Lett. 2011;107(6):060601.
9. Lewis JT, Russell R, Toomey F, McGurk B, Crosby S, Leslie I. *Practical connection admission control for ATM networks based on on-line measurements.* Comput Commun. 1998;21(17):1585–1596.
10. Rasmussen CE. *Gaussian processes in machine learning.* In: Summer school on machine learning. Springer; 2003. p. 63–71.
